# *OsBT1* encodes an ADP-glucose transporter involved in starch synthesis and compound granule formation in rice endosperm

**DOI:** 10.1038/srep40124

**Published:** 2017-01-05

**Authors:** Sanfeng Li, Xiangjin Wei, Yulong Ren, Jiehua Qiu, Guiai Jiao, Xiuping Guo, Shaoqing Tang, Jianmin Wan, Peisong Hu

**Affiliations:** 1 State Key Laboratory of Rice Biology, China National Rice Research Institute, Hangzhou, 310006, China; 2 National Key Facility for Crop Resources and Genetic Improvement, Institute of Crop Science, Chinese Academy of Agricultural Sciences, Beijing 100081, P.R. China

## Abstract

Starch is the main storage carbohydrate in higher plants. Although several enzymes and regulators for starch biosynthesis have been characterized, a complete regulatory network for starch synthesis in cereal seeds remains elusive. Here, we report the identification and characterization of the rice *Brittle1 (OsBT1*) gene, which is expressed specifically in the developing endosperm. The *osbt1* mutant showed a white-core endosperm and a significantly lower grain weight than the wild-type. The formation and development of compound starch granules in *osbt1* was obviously defective: the amyloplast was disintegrated at early developmental stages and the starch granules were disperse and not compound in the endosperm cells in the centre region of *osbt1* seeds. The total starch content and amylose content was decreased and the physicochemical properties of starch were altered. Moreover, the degree of polymerization (DP) of amylopectin in *osbt1* was remarkably different from that of wild-type. Map-based cloning of *OsBT1* indicated that it encodes a putatively ADP-glucose transporter. *OsBT1* coded protein localizes in the amyloplast envelope membrane. Furthermore, the expression of starch synthesis related genes was also altered in the *osbt1* mutant. These findings indicate that *OsBT1* plays an important role in starch synthesis and the formation of compound starch granules.

Starch is the main storage carbohydrate in higher plants and not only is the primary source of calories in human diet but also has great value in industrial applications. Because its starch content, rice (*Oryza sativa* L.) is one of the most important staple foods for more than half of the world’s population. Starch synthesis and accumulation occur in the amyloplasts that exist in tubers, cotyledons, and endosperms, and is also an important determinant for crop yield^1^,^2^.

Starch comprises two major components, amylose and amylopectin. A series of key enzymes involved in starch biosynthesis have been identified. ADP-glucose (ADPG) is the substrate for starch synthesis, which is converted from glucose-1-phosphate by ADP-glucose pyrophosphorylase (AGPase). Granule-bound starch synthase I (GBSSI), which is encoded by the *Waxy* gene is required for the synthesis of amylose^3^,^4^. On the other hand, a series of starch synthases (SSI, SSII, SSIII, and SSIV), starch branching enzymes (BEI and BEII), and starch debranching enzymes (ISA and Pullulanase) control the synthesis of amylopectin^5^,^6^,^7^. Mutants defective in these genes show abnormal characteristics of endosperm stored starch. For instance, mutations in *OsAGPL2* and *OsAGPS2b* (encoding a large and small AGPase subunits, respectively) cause a shrunken endosperm, due to a significant reduction in starch synthesis^8^. Loss-of-function mutations of the rice *GBSSI (Waxy*) gene produce an amylose-free endosperm^9^,^10^. A deficiency of *SSIIIa* leads to abnormal amylose content, amylopectin structure, and starch granule properties^11^,^12^. Loss-of-function mutation of *ISA1* produces a sugary endosperm and a dramatic alteration of polyglucan structure from amylopectin to phytoglycogen^13^,^14^. Mutation of *BEIIb* in the *amylose-extender (ae*) mutant generates a chalkiness endosperm and alterations in the structure of amylopectin and in the gelatinization properties of starch granules^15^.

Besides these key enzyme genes, several regulators are known to indirectly regulate starch biosynthesis in rice endosperm. For instance, FLO2, a tetratricopeptide (TPR) domain-containing protein, regulates the expression of genes involved in the production of storage starch and storage proteins in the endosperm *via* interaction with a bHLH protein^16^. *FLO6*, encoding a CBM48 domain-containing protein, is involved in compound granule formation and starch synthesis putatively through interaction with ISA1^17^. *Grain Incomplete Filling 1 (GIF1*) encodes a cell-wall invertase that regulates sucrose unloading, and the mutation of *GIF1* leads to significantly reduced starch content and grain weight^18^. Mutant for the *OsAlaAT1* gene, encoding an alanine-aminotransferase, also displays altered amylose content and amylopectin structure^19^. The bZIP transcription factor OsbZIP58 can regulate the expression of six starch-synthesizing genes by directly binding to their promoters^20^. *Rice Starch Regulator (RSR1*), encoding an AP2/EREBP family transcription factor, negatively regulates the expression of type I starch synthesis genes, and *RSR1* deficiency results in enhanced expression of starch synthesis genes in seeds^21^. Despite all these advances, the identification of novel genes involved in starch synthesis is necessary to understand the molecular network underlying starch synthesis and seed quality formation in rice.

As the substrate for starch synthesis, ADPG needs to be transported into amyloplasts from the cytosol by an ADPG transporter located in the envelope membrane of amyloplasts. Sullivan *et al*. first proposed BT1 proteins as ADPG transporters in cereal endosperms^22^. These BT1 proteins belong to the mitochondrial carrier family (MCF)^23^,^24^. Although MCF proteins are presumed to be targeted to the mitochondrial inner membrane in plants^23^, some also occur in peroxisomes, glyoxysomes, plasma membrane, and plastids and are involved in the transport of diverse solutes across these organelle membranes^25^,^26^,^27^,^28^,^29^. Transport studies have shown that maize ZmBT1, barley HvNST1, and wheat TaBT1 are able to transport ADPG in counter exchange with ADP^30^,^31^,^32^. OsBT1 corresponds to the rice orthologue^32^ which may also function to transport ADPG.

In this study, we characterize a white-core endosperm mutant, *osbt1*. Map-based cloning showed that *OsBT1* encodes an ADPG transporter which locates in the amyloplast envelope membrane and functions in the regulation of starch biosynthesis and compound starch granule formation during rice seed development.

## Results

### The rice *osbt1* mutant has a defect in endosperm appearance and grain weight

**Figure 1:**
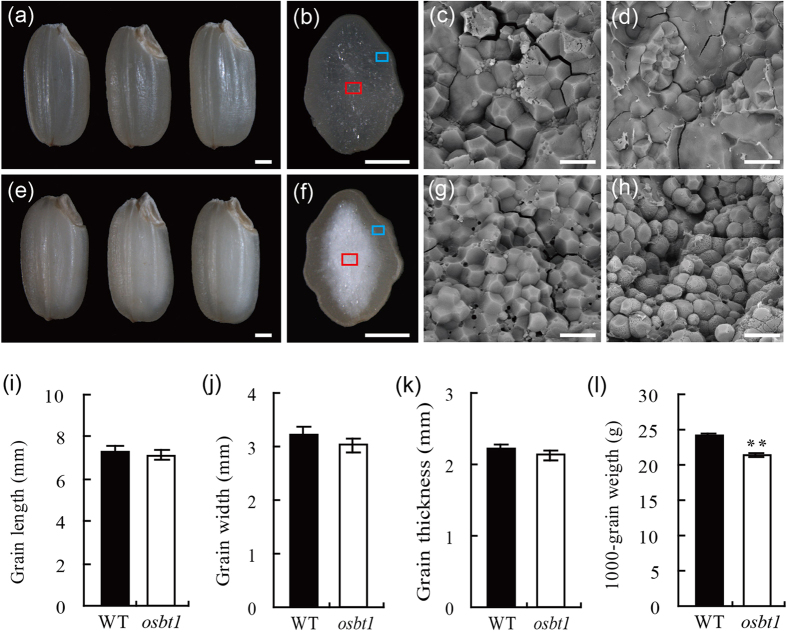
Characterization of the *osbt1* mutant. (**a**,**e**) Appearance of mature seeds of wild-type (WT) (**a**) and *osbt1* (**e**); (**b**,**f**) Cross-sections of mature seeds of WT (**b**) and *osbt1* (**f**); (**c**,**g**) SEM analysis of the periphery area of mature endosperm of WT (**c**) and *osbt1* (**g**). The periphery areas shown are indicated as blue squares in (**b**,**f**); (**d**,**h**) SEM analysis of the central area of mature endosperm of WT (**d**) and *osbt1* (**h**). The central areas shown are indicated as red squares in (**b**,**f**); Scale bars: 1 mm in (**a**,**b**,**e**,**f**); 20 μm in (**c**,**d**,**g**,**h**); (**i**–**l**) Quantification of WT and *osbt1* seed size and grain weight including grain length (**i**), grain width (**j**), grain thickness (**k**) and 1000-grain weight (**l**). Data are given as means ± SD from three replicates. Statistical comparisons were performed using Student’s *t*-test; all data were compared with WT (*P < 0.05, **P < 0.01).

Numerous mutants with chalky endosperm induced by tissue culture were screened and a mutant with white core was obtained and later named *osbt1*. The endosperm of *osbt1* was opaque and presented a white-core in the centre region of the seed, while the periphery region was translucent compared to that of wild-type (Fig. 1a,b,e,f). Scanning electron microscopy (SEM) analysis of transverse sections indicated that the starch grains (SGs) in both central and periphery regions of the wild-type endosperm cells were polyhedral and densely packed (Fig. 1c,d). The SGs in the endosperm peripheral cells of the *osbt1* mutant were similar to those of the wild-type (Fig. 1g), while those in the inner endosperm cells of the mutant were loosely packed with spherical or oval and smooth surface (Fig. 1h). Quantification of seed size showed no significant differences between wild-type and *osbt1* in terms of seed length, width, and thickness (Fig. 1i–k), though 1000-grain weight in *osbt1* was significantly decreased compared to wild-type (Fig. 1l). Besides the endosperm defect and lower grain weight, *osbt1* plants did not display any visible differences from wild-type during the whole growth period (Fig. S1).

### The compound starch granules in *osbt1* endosperm cells are abnormal

**Figure 2:**
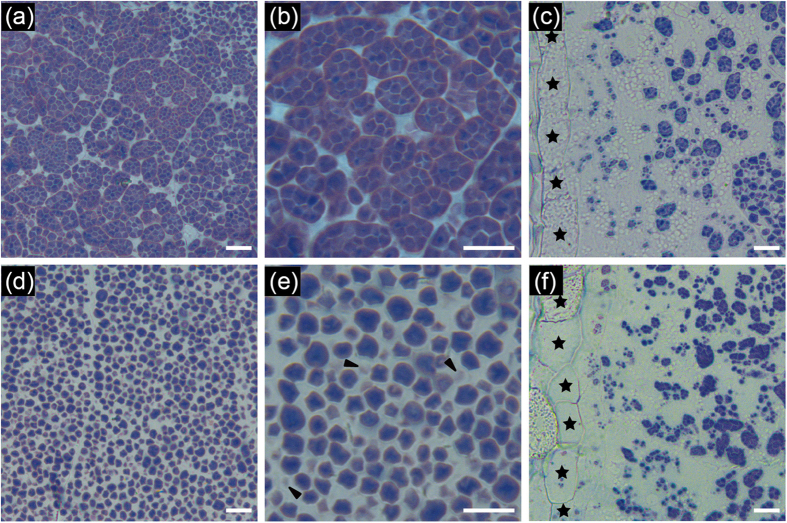
Abnormal compound granules formation in *osbt1* seeds. Semi-thin sections of WT (**a–c**) and *osbt1* mutant (**d–f**) endosperm at 9 DAF. (**a,b**,**d,e**) The central region of endosperm cells; (**c,f**) The peripheral region of endosperm cells. Stars indicate aleurone cells in (**c**,**f**). Arrowheads in (**e**) indicate smaller, abnormal starch granules in cytosol. Scale bars: 10 μm.

Semi-thin sections of developing endosperms at 9 days after fertilization (DAF) were prepared to compare the formation of compound starch granules in endosperm cells between *osbt1* and wild-type (Fig. 2). The results showed that the amyloplasts in wild-type endosperm cells were composed of several polyhedral, sharp-edged, and easily separable granules, forming well developed compound starch granules. The entire central region of the wild-type endosperm was almost filled with compound starch granules (Fig. 2a,b). However, abundant single starch granules were observed in the centre of the *osbt1* mutant endosperm. In addition, some abnormal weakly stained granules were also observed in the mutant (Fig. 2d,e). By contrast, the endosperm peripheral cells of *osbt1* were similar to those of wild-type, both having fewer well developed compound starch granules and some immature amyloplasts (Fig. 2c,f).

**Figure 3:**
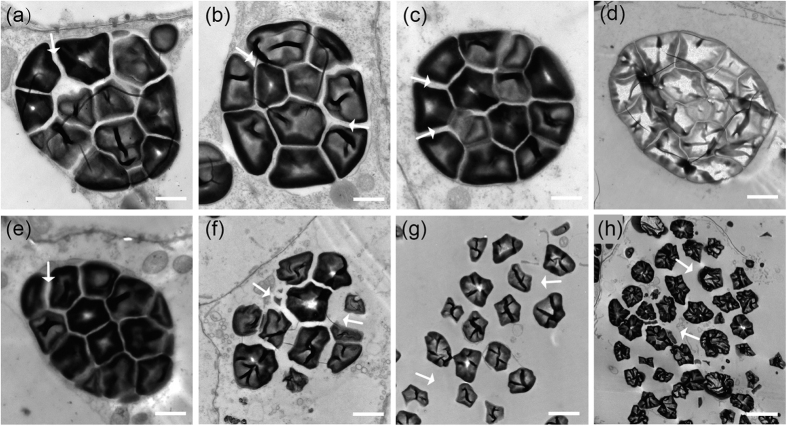
Electron micrographs depicting amyloplast development in WT (**a–d**) and the *osbt1* mutant (**e,h**) endosperms. (**a,e**) A representative amyloplast in peripheral endosperm cells of WT and *osbt1* at 6 DAF; (**b–d**,**f–h**) An amyloplast in central endosperm cells of WT and *osbt1* at 6 (**b,f**), 9 (**c,g**), and 12 DAF (**d,h**), respectively. White arrows indicate the stroma inside the amyloplast. Bars: 2 μm.

Transmission electron microscope (TEM) analysis was further carried out to observe the development of compound starch granules. The amyloplasts in the endosperm peripheral cells of both wild-type and *osbt1,* and the central endosperm cells of wild-type were filled with about a dozen of polyhedral starch granules and the space between the starch granules was gradually occupied during seed development, to eventually form normal compound starch granules (Fig. 3a–e). However, in the *osbt1*, the amyloplasts were disintegrated at early developmental stages and the starch granules were dispersive through the central endosperm cells (Fig. 3f–h), which was consistent with the results observed in the semi-thin sections (Fig. 2d,e).

### Starch physicochemical properties are altered in the *osbt1* mutant

**Figure 4:**
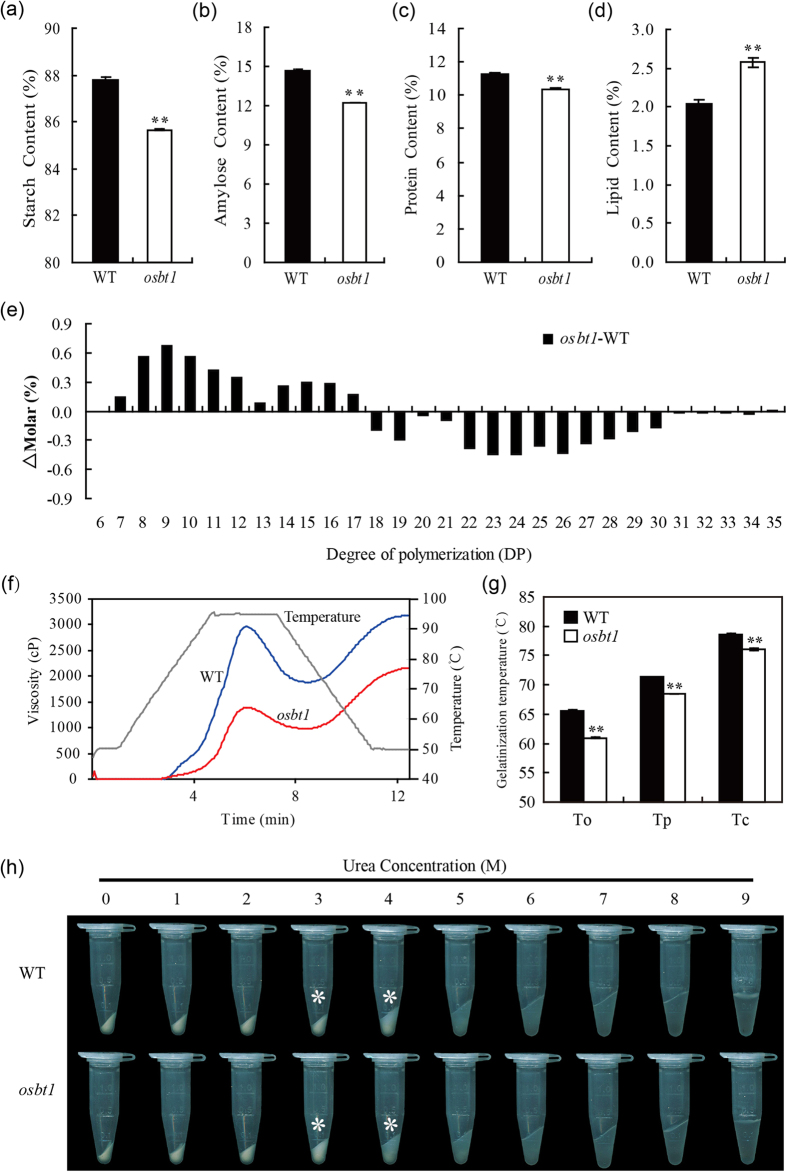
Seed properties and starch physicochemical characteristics in the *osbt1* mutant. (**a–d**) Quality trait parameters of the WT and *osbt1*. Values are means ± SD (n = 3). The asterisks indicate statistical significance between WT and the mutant, as determined by a Student’s *t*–test (*P < 0.05; **P < 0.01). (**e**) Differences in the amylopectin chain length distributions between the WT and *osbt1*. (**f**) Pasting properties of endosperm starch of WT (blue line) and *osbt1* (red line). The viscosity value at each temperature is the average of three replicates. The gray line indicates the temperature changes during the measurements. (**g**) Gelatinization temperature of endosperm starch. To, Tp, and Tc represent the onset, peak, and conclusion gelatinization temperatures, respectively. All data are presented as means ± SD from three replicates. Two-tailed unpaired *t*-tests indicate the significant differences: *P < 0.05, **P < 0.01. (**h**) Gelatinization characteristics of starch from *osbt1* mutant seeds. Starch powder was mixed with different concentrations (1–9 M) of urea solution. The most significant difference was observed for 3–4 M urea.

An analysis of the physicochemical characteristics of the starch was performed in wild-type and the *osbt1* mutant. The total starch and amylose contents were significantly decreased in *osbt1* compared to wild-type (Fig. 4a,b). The protein content was lower, while the lipid content was clearly higher than those in the wild-type (Fig. 4c,d). To further examine the fine structure of amylopectin, the chain length distribution of amylopectin was also determined. Compared to wild-type, the proportion of short chains with degree of polymerization (DP) values between 7 and 17 were elevated in the mutant, whereas the proportion of intermediate chains with DP values between 18 and 30 were decreased (Fig. 4e).

The starch pasting properties were analyzed with a rapid visco analyzer (RVA; Fig. 4f). The starch pasting characteristics in wild-type showed distinct parameters represented by a peak viscosity, a fast viscosity increase when the temperature decreased and a high final viscosity value. The viscosity pattern of the *osbt1* starch presented a trend similar to wild-type, but was maintained at lower levels, with a final viscosity of 68% of that of wild-type. The thermal gelatinization temperature of endosperm starch from wild-type and *osbt1* were analyzed and the result showed that the onset (To), peak (Tp), and conclusion (Tc) temperatures of gelatinization of endosperm starch in the *osbt1* mutant were all about 3 °C lower than those in the wild-type (Fig. 4g). The gelatinization properties of the SGs were also examined by measuring solubility in urea solutions^15^. Various concentrations (0–9 M) of urea solutions were added to powdered starch. The result showed that the gelatinization characteristics of *osbt1* starch have most significant differences in 3–4 M urea from that of wild-type (Fig. 4h). In summary, the starch physicochemical characteristics of *osbt1* were obviously different from those of the wild-type.

### Map-based cloning and complementation of the *OsBT1* gene

**Figure 5:**
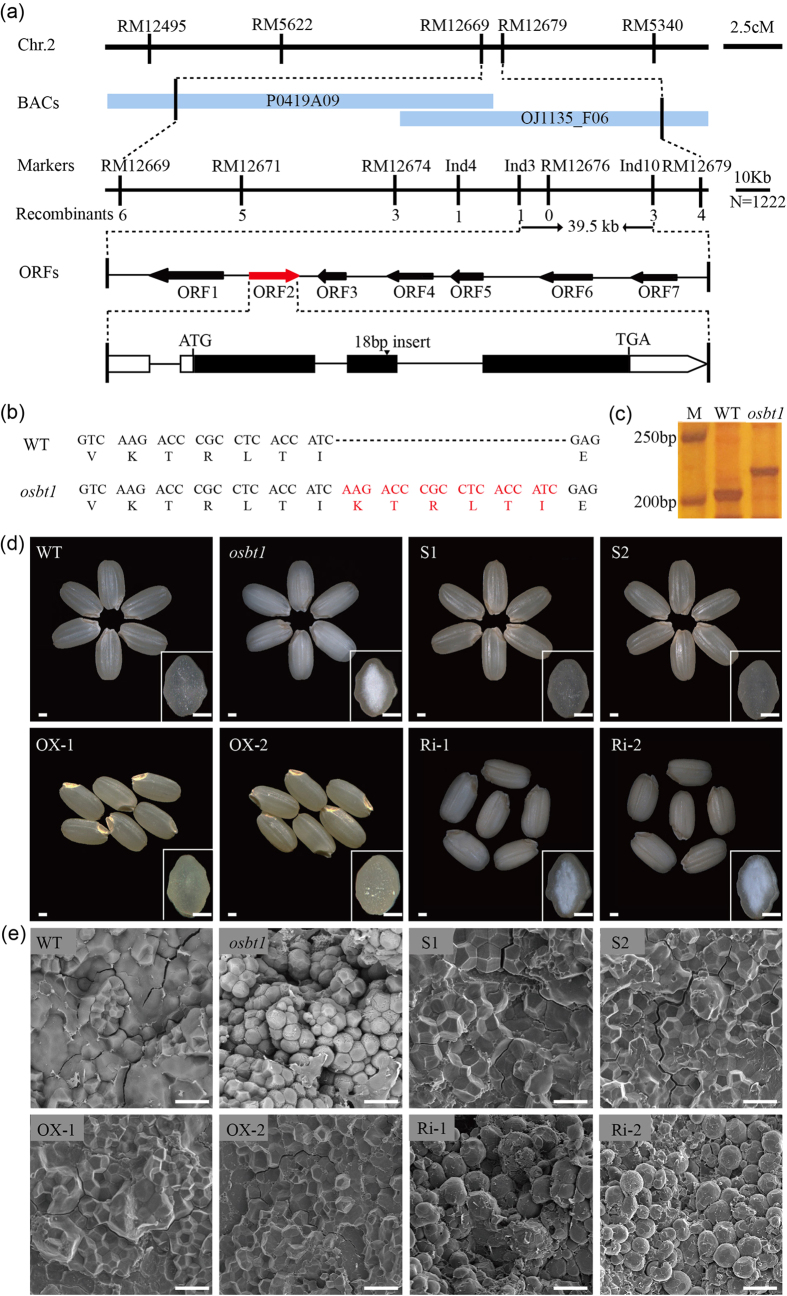
Map-based cloning, complementation of the *osbt1* mutant and phenotype of transgenic lines. (**a**) Fine mapping of the *OsBT1* locus. The *OsBT1* locus was mapped to a 39.5 kb region by markers Indel1-3 and Indel1-10 on chromosome 2 (Chr.2), which contained seven predicted genes. The molecular markers and the number of recombinants are shown. cM, centimorgan; ORF, open reading frame. (**b**) Gene structure and mutation site in *osbt1*. The cDNA sequence comparison shows the 18 bp insertion in *osbt1* leading to six amino acid addition. (**c**) Identification of the 18 bp insertion between the WT and *osbt1* using an Indel marker. M, Marker. (**d**) Complementation of the *osbt1* mutation in transgenic lines (S1–S2) and overexpressing the *OsBT1* in *osbt1* (OX-1, OX-2) showing the completely restored WT seed appearance (**d**) and the starch granules morphology (**e**). RNAi of *OsBT1* in WT genotype produce abnormal seed appearance. RNAi seeds (Ri-1, Ri-2) became chalkiness (**d**), and the starch granules became abnormal (**e**). Insets in (**d**) represent the transverse sections of representative grains. Bars: 1 mm in (**d**); 20 μm in (**e**).

Map-based cloning was carried out to identify the gene affected in the mutant. An F_2_ segregating population was created by crossing the *osbt1* mutant (*japonica*) with NJ-11 (*indica*). The mutated locus was first mapped to a short arm in chromosome 2 between the simple sequence repeat (SSR) markers RM12669 and RM12679. Based on three SSR markers and three newly-developed insertion/deletion (Indel) markers, the *osbt1* locus was further narrowed down to a 39.5-kb region between markers Ind3 and Ind10 by using a total of 1,222 recessive homozygous individuals. Seven open reading frames (ORFs) were included in this region (Fig. 5a and Table S1). Sequence analysis revealed that there was a 18-bp repeat insertion in ORF2 (*Os02g0202400*) in the mutant, which lead to a six amino acid increase in the encoded protein compared to the wild-type sequence (Fig. 5b,c). The three-dimensional structures of OsBT1 in wild-type and *osbt1* were predicted using Protein Homology/analogy Recognition Engine V 2.0 (Phyre2) web portal, the result showed that the OsBT1 protein cannot be normally folded due to six amino acids added in *osbt1* (Fig. S2).

To test whether *Os02g0202400* corresponds to the candidate *OsBT1* gene, a vector bearing the *OsBT1* genomic sequence including its native promoter and a vector bearing the *OsBT1* coding region driven by the *Actin1* promoter were constructed and introduced into the *osbt1* mutant. The expression of *OsBT1* in complementary and overexpressing lines was significant higher than that in the wild-type (Fig. S3). Seeds produced by independent positive transgenic T_1_ lines showed to the wild-type appearance restored (Fig. 5d). The 1000 brown grains weight in complementary and overexpressing lines also restored to wild-type level and the brown grain length, width, and thickness had no significant differences compared to the wild-type (Fig. S4). The SGs in endosperm cells of these positive transgenic lines also presented the polyhedral and densely packed granules morphology, similar to those of wild-type (Fig. 5e). A further validation was obtained by analyzing *Os02g0202400* RNAi knock-down plants (Ri) generated in a wild-type genetic background which showed reduced expression of *OsBT1* (Fig. S3). Grains harvested from the independent Ri transgenic T_1_ lines showed the *osbt1* mutant appearance (Fig. 5d). The 1000 brown grains weight in Ri lines significantly decreased and the brown grain length, width, and thickness had no significant differences compare to the wild-type (Fig. S4). Moreover, the SGs morphology in the Ri endosperm cells was similar to those of the *osbt1* mutant (Fig. 5e). All these results demonstrated that *Os02g0202400* is the gene responsible for the *osbt1* phenotypes.

### Expression pattern and subcellular localization of *OsBT1*

**Figure 6:**
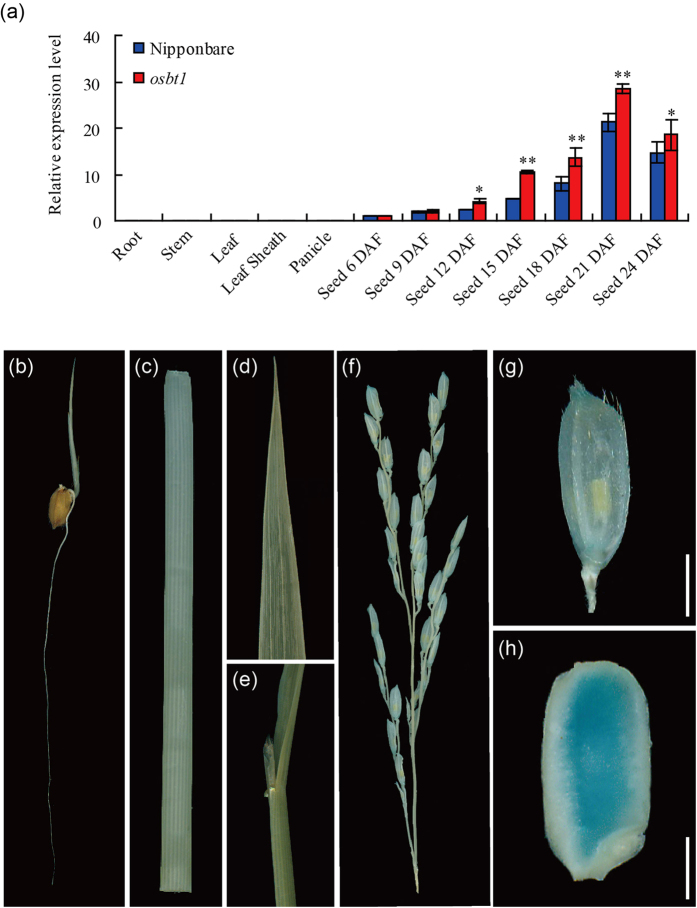
Expression pattern of *OsBT1.* (**a**) qRT-PCR analysis of *OsBT1* expression level in various tissues and in developing endosperms of the WT and *osbt1*. Values are means ± SD (n = 3). The asterisks indicate statistical significance between the WT and the mutant, as determined by a Student’s *t*-test (*P < 0.05; **P < 0.01). (**b–h**) GUS expression in root (**b**), steam (**c**), leaf (**d**), leaf sheath (**e**), panicle (**f**), spikelet (**g**) and brown rice (**h**) driven by the *OsBT1* promoter. Bars: 2 mm.

The temporal and spatial expression patterns of *OsBT1* were analyzed by quantitative real-time PCR (qRT-PCR). The results showed that *OsBT1* was specifically expressed in seeds and that its relative expression level gradually increased after fertilization, with a maximum expression level at 21 DAF (Fig. 6a). Moreover, GUS histochemical assay showed that the *OsBT1* promoter directed the GUS reporter gene to express predominantly in the endosperm but not in roots, stems, leaves, leaf sheaths, and panicles (Fig. 6b–h).

**Figure 7:**
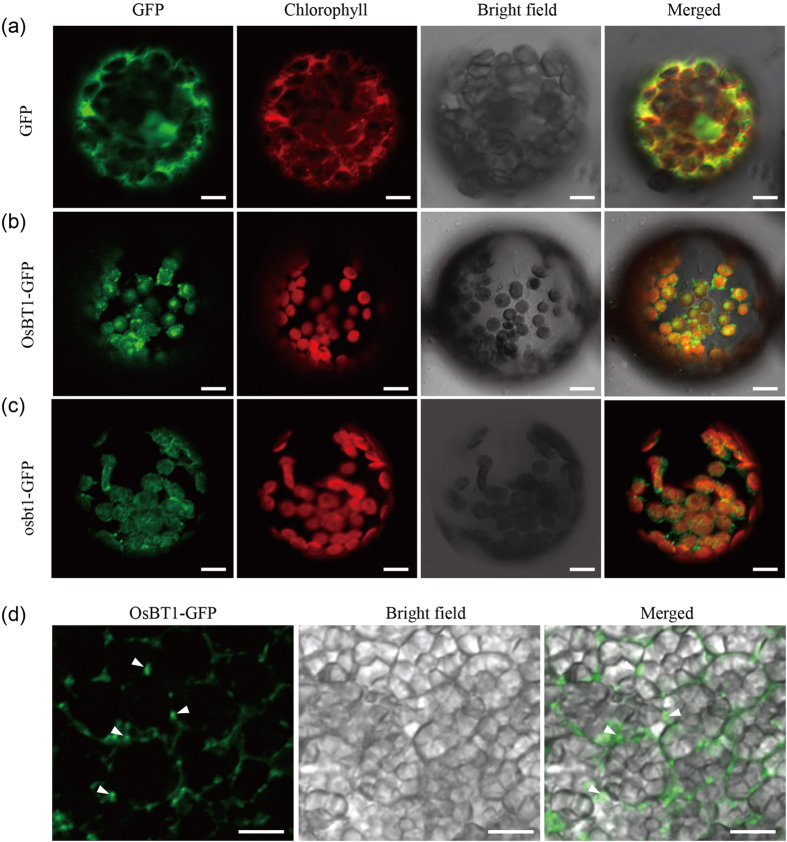
Subcellular localization of OsBT1. (**a**) Free GFP used as a control. (**b**,**c**) Full-length coding region fusion protein OsBT1-GFP (**b**) and osbt1-GFP (**c**) in front of the GFP. 16 h after transformation, tobacco protoplasts were observed using a confocal laser scanning microscope. Green fluorescence signals (GFP), red chlorophyll autofluorescence (red), bright-field images, and an overlay of green and red signals are shown in each panel. (**d**) OsBT1-GFP fusion protein was expressed in rice under the control of the maize *UBIQUITIN1* promoter and then the localization of OsBT1-GFP in developing endosperm was observed by confocal laser scanning microscope. The outer limit envelope of the amyloplasts is indicated by white arrowhead. Scale bars: 10 μm.

Subcellular localization of the OsBT1 protein was carried out using two GFP fusions, OsBT1-GFP and osbt1-GFP that were transiently expressed in tobacco protoplasts. The free GFP control protein was widely expressed in the cytoplasm and nuclei (Fig. 7a), whereas OsBT1-GFP and osbt1-GFP both co-localized with the chlorophyll autofluorescence signals of the plastids (Fig. 7b,c). Another vector harboring OsBT1-GFP driven by the maize *UBIQUITIN1* promoter (*Ubi*) was generated and transformed into the Kitaake rice cultivar. Laser scanning confocal microscope showed that OsBT1-GFP was exclusively localized at the amyloplast envelope membranes in endosperm cells (Fig. 7d). All these results demonstrated that OsBT1 localizes to the envelope membrane of amyloplasts, and that the mutation in *osbt1* did not affect the protein localization.

### The expression of starch synthesis related genes is changed in the *osbt1* mutant

**Figure 8:**
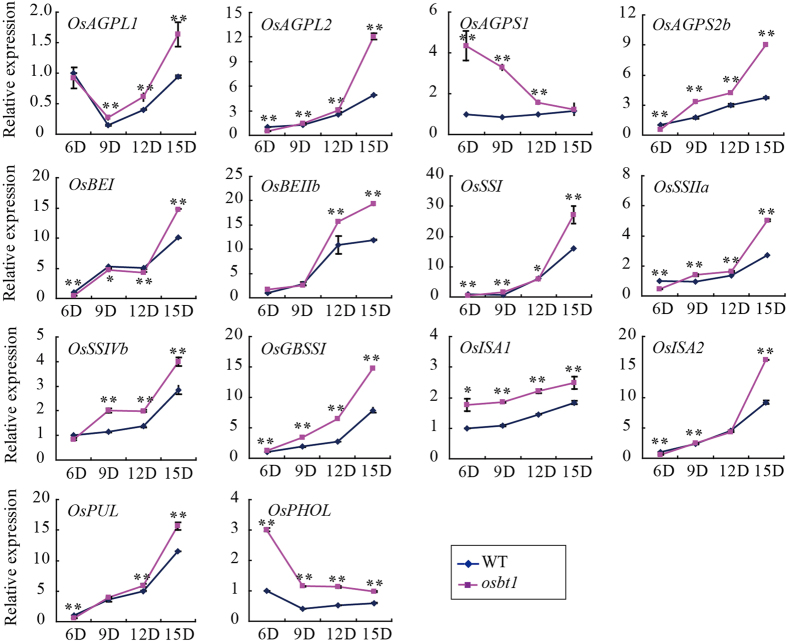
Expression profiles of rice starch synthesis genes during seed development in WT and *osbt1*. Total RNA extracted from developing seeds at 6, 9, 12 and 15 DAF was used for qRT-PCR analysis. The expression of each gene in the 6 DAF WT seeds was set as reference value of 1. All data are shown as means ± SD from three replicates. The asterisks indicate statistical significance between the WT and the mutant, as determined by a Student’s *t*-est (*P < 0.05; **P < 0.01).

The expression pattern of starch synthesis related genes was examined in wild-type and *osbt1* endosperms at 6, 9, 12 and 15 DAF *via* qRT-PCR (Fig. 8). On the whole, results showed that expression of the 14 starch synthesis related genes was higher in *osbt1* than that in the wild-type. The expression levels of *OsGBSSI, OsISA1*, and *OsPHOL* were always higher in the mutant compared to the wild-type from 6 to 15 DAF. The transcript levels of other genes including two AGP genes (*OsAGPL2,* and *OsAGPS2b*), and five amylopectin synthesis related genes (*OsSSI, OsSSIIa, OsSSIVb, OsISA2*, and *OsPUL*) were lower at 6 DAF, but were elevated at 9 to 15 DAF compared to wild-type. The expression levels of *OsAGPL1* and *OsBEIIb* were higher in *osbt1* than in the wild-type, but no significant differences were detected at 6 or 9 DAF. *OsAGPS1* expression was higher in *osbt1* than in the wild-type at the early grain filling stage (6–12 DAF), but maintained a similar expression level at 15 DAF. The expression pattern of the *OsBE1* gene was opposite to *OsAGPS1*, which was lower than in the wild-type at the early grain filling stage (6–12 DAF), but higher at 15 DAF (Fig. 8). Additionally, a zymogram analysis indicated that the activities of Pho (Pho1 and Pho2) and SSI were increased in the *osbt1* mutant while the activity of SSIIIa has no significant differences in the mutant compared to wild-type (Fig. S5).

## Discussion

Starch biosynthesis is a complex metabolic process and the molecular regulatory machinery remains largely unknown. Characterization of new rice endosperm-defective mutants is useful to further elucidate the starch synthesis process. Previous studies have identified at least 29 endosperm-defective mutants, including six chalky endosperm mutants (*flo4, flo5, rsr1, gif1, bzip58*, and *nfyb1*)^12^,^18^,^20^,^21^,^33^,^34^, 16 floury endosperm mutants (*flo1-flo3, ae, pdil1-1, gpa1* to *gpa3, osagpl2–3, flo6, ssg4, osalaat1, flo7, ssg6, pfp*, and *ss3a/ss4b*)^16^,^17^,^18^,^19^,^35^,^36^,^37^,^38^,^39^,^40^,^41^,^42^,^43^,^44^,^45^,^46^,^47^, four shrunken endosperm mutants (*isa1/sugary1, osagpl2, osagps2*, and *pho1*)^8^,^14^,^48^, and three dull/waxy endosperm mutants (*dull1, dull3*, and *waxy*)^10^,^49^,^50^. In addition, the quantitative trait locus *Chalk5* was also identified to influence grain chalkiness in rice^51^. Most of these mutants show decreased amylose content and changed amylopectin structure, and some also exhibit slower grain-filling and decreased 1000-grain weight.

In this study, we isolated and characterized the *osbt1* mutant, which exhibits white-core endosperm, abnormal compound starch granules formation, decreased starch content, altered amylopectin structure and physiochemical properties and significant reduced final grain weight. These data indicate that starch synthesis in *osbt1* mutant endosperm is seriously affected. In addition, the morphology of the compound starch granules in amyloplasts of some endosperm-defective mutants has also been reported to be abnormal. For example, mutations in *SSG4* and *SSG6* develop enlarged amyloplasts^37^,^45^, while *flo6* endosperm contains smaller and abnormal compound granules^17^. In our case, the amyloplasts were disintegrated at early developmental stages and the starch granules were dispersive (not compound) in the central endosperm cells of *osbt1* (Fig. 2). We propose that *OsBT1* plays a crucial role in maintaining the stability of the structure of the amyloplast envelope membrane.

Usually, regulators of starch synthesis affect the expression level of genes encoding key enzymes of the starch synthesis pathway. For example, *RSR1* negatively regulates the expression of type I starch synthesis genes (i.e. the expression of most starch synthesis genes is higher in the *rsr1* mutant and repressed in *RSR1*-overexpressing lines)^21^. When another starch biosynthesis regulator, *OsbZIP58,* was inactivated, the expression of *OsAGPS2b, OsAGPL2, OsSSI, OsSSIIIa, OsSSIVb, OsBEIIb*, and *OsISA2* was distinctly up-regulated, while that of *OsAGPL3, OsPHO1, Wx*, and *SBE1* was down-regulated, leading to a reduced content of total starch and amylose. Also, the fine structure of amylopectin is altered in the *osbzip58* mutant^20^. When *Chalk5*, which encodes a vacuolar H^+^-translocating pyrophosphatase influencing grain chalkiness in rice, is over-expressed, the transcript levels of eight genes involved in starch metabolism are significant elevated in seeds at 5 DAF, implying a feedback regulation of *Chalk5* expression in controlling grain chalkiness^51^. In our study, the expression of several key starch synthesis genes was significantly enhanced in the endosperm of *osbt1* (Fig. 8). Expression changes of some starch synthesis related genes may alter the structure of amylopectin that further changes the crystalline structure of the starch granule, consequently affecting the physicochemical properties of starch. For example, SSI appears to be primarily responsible for synthesizing shorter chains of amylopectin^52^, whereas BEIIb functioned in the branching of short chains, as revealed by *in vitro* analysis^53^. So the increased short chains in *osbt1* may result from enhanced *OsSSI* and *OsBEIIb* expression. In our study, the increased short chains lead to a lower gelatinization temperature (Fig. 4g), which was consistent with what was observed in the *sbe1* mutant^15^,^54^. For these starch synthesis related genes located downstream of *OsBT1*, mutation of *OsBT1* results in lack of enough substrate for starch synthesis, so the elevated expression of starch synthesis related genes likely involve a physiological feedback regulation, which is similar to that in the *osbzip58* and *rsr1* mutant^20^,^21^.

As the predominantly product of photosynthesis, sucrose occurs in source leaves, which is then transported into endosperm cells to be used as carbon source for starch synthesis. Sucrose is divided and converted into ADPG, G6P, and Glc and then imported into amyloplasts by their corresponding transporters (Fig. S6). The glucose 6-phosphate/phosphate translocator (GPT), which is located on the endosperm plastid envelope membranes, is responsible for transporting G6P into plastids^55^. The plastidic glucose transporter (pGlcT) located in the envelope of chloroplasts has a role in the export of glucose, and also is located in the envelope of endosperm amyloplast with the role to import glucose^56^. As substrate of starch synthesis in endosperm cells, ADPG needs to be transported into the amyloplast by the ADPG transporter located in its envelope from the cytosol. The designation of BRITTLE-1 (BT1) protein as the ADPG transporter in cereal endosperms was first proposed by Sullivan *et al*. (1991)^22^, and then characterized in maize, barley, and wheat^30^,^31^,^32^. In this study, the rice *OsBT1* gene was identified using a forward genetics approach. Subcellular localization analysis showed that OsBT1 is located to the envelope membrane of amyloplasts. Thus, we proposed that OsBT1 is the ADPG transporter in rice endosperm cells. To validate our hypothesis, we carried out import studies using [^14^C] ADPG into *Escherichia coli* cells harboring recombinant OsBT1, however this assumption was also recently confirmed by Cakir *et al*.^57^. Together, these data demonstrate that OsBT1 is the rice ADPG transporter which transports the substrate ADPG into amyloplasts from the cytosol of endosperm cells.

In summary, our results indicate that *OsBT1* has an essential role in starch synthesis and compound starch granule formation. Ongoing efforts are still needed to identify new regulatory steps of starch biosynthesis as means to aid breeders and biotechnologists to improve quality in crops.

## Materials and Methods

### Plant materials and growth conditions

The *osbt1* mutant was generated from *Oryza sativa japonica* cv. Nipponbare induced by tissue culture *in vitro*. An F_2_ population was produced by crossing the *osbt1* mutant with an *indica* rice cv. Nanjing11 (NJ-11) for gene mapping. The rice plants were grown under natural conditions at China National Rice Research Institute experimental field plots, in Hangzhou, China.

### Microscopy

The brown rice of wild-type and the *osbt1* mutant was cut transversely with the back of a knife, and the ruptured transverse surface was coated with gold to prepare samples. The ruptured transverse surface was observed by scanning electron microscope (SEM) which was performed as described previously using a HITACHI S-3400N scanning electron microscope (http://www.hitachi-hitec.com) 33. For analyzing the development of compound starch granules, transverse sections (approximately 1 mm in thickness) of wild-type and *osbt1* endosperms at 9 DAF were used to make samples of semi-thin sections. Samples were treated as described by Peng *et al*.^17^. Semi-thin sections (800 nm) were stained with I_2_-KI for 5 s and subsequently examined under a light microscope (Nikon Eclipse 80i; http://www.nikon.com). For the ultrastructure observation of amyloplasts, developing seeds (6–12 DAF) were fixed over 12 h in 0.2 M phosphate buffer (PH 7.2) with 2.5% glutaraldehyde. Samples were treated as described by Takemoto *et al*.^58^ and sectioned using an ultramicrotome (Power Tome-XL; RMC, http://www.rmcproducts.com). A transmission electron microscope (H-7650; Hitachi, http://www. hitachi.com) was used for observation.

### Physicochemical properties of rice grains

The total starch content of the rice flour was measured according to the manufacturer’s protocol, with a starch assay kit (Megazyme, Wicklow, Ireland, http://www.megazyme.com/). Amylose content was determined as described by Liu *et al*.^59^, and the lipid and protein contents in the seeds of *osbt1* mutant and wild-type were measured following the method described by Kang *et al*.^33^. To determine the starch pasting properties, 3 g of milled rice flour (0.5 mm or less, 14% moisture basis) was transferred into a vessel containing 25 ml of distilled water. The sample was mixed and measured with a Rapid Visco Analyzer (RVA Techmaster, Newport Scientific, Narrabeen, Australia), applying the temperature program described by the America Association of Cereal Chemists, Approved Method 61–02.01 (AACC, 1995). To determine the chain length distribution of amylopectin, 5 mg of rice powder was digested with *Pseudomonas amyloderamosa* isoamylase (Megazyme) and then analyzed by capillary electrophoresis (PA800 plus pharmaceutical analysis system, Beckman Coulter, USA; http://www.beckmancoulter.com/). For the gelatinization temperature analysis, 5 mg of rice powder was placed in an aluminum sample cup, mixed with 10 μl of distilled water, and sealed, and then the samples were analyzed by a differential scanning calorimeter (DSC 1; METTLER-TOLEDO). The heating rate was 10 °C min^−1^ over a temperature range of 35 °C to 100 °C. The swelling modes of endosperm starch in urea solution were measured according to a previous report^15^.

### Mapping of the *OsBT1* gene

Seeds which have similar white-core mutant phenotype were selected from the F_2_ population which derived from a cross between the *osbt1* mutant and an *indica* rice cv. NJ-11. Then these white-core seeds were grown until seedling stage for DNA extraction. Twenty-two F_2_ plants with mutant phenotype and more than 160 polymorphism simple sequence repeat markers evenly distributed over the whole genome were selected to identify markers linked to the *OsBT1* locus. Further molecular markers were developed based on the nucleotide polymorphisms in the corresponding regions between the Nipponbare and NJ-11 (Table S2). A total of 1,222 recessive individuals with the mutant phenotype were selected from the F_2_ population to fine-map the *OsBT1* locus.

### Plasmid construction and rice transformation

The genomic fragment of the *OsBT1* gene, included its native promoter from Nipponbare was amplified by PCR (all primers used for plasmid construction are listed in Table S2) and then subcloned into the binary vector pCAMBIA2300 to generate the complementation vector. A vector bearing the *OsBT1* coding region driven by Actin1 promoter was constructed and then sub-cloned into the binary vector pCAMBIA2300 to generate the overexpressing vector. A specific sequence from the *OsBT1* cDNA from Nipponbare was cloned into the binary vector LH-FAD2-1390RNAi under the control of the maize *UBIQUTIN1* promoter to obtain the RNAi vector. The resulting plasmids were introduced into *osbt1* and the wild-type plants, respectively, by the *Agrobacterium tumefaciens*-mediated method. The genotype of transgenic plants was determined by PCR amplification of the specific transgenic fragment. A 2.1 kb sequence of *OsBT1* promoter region was isolated by PCR amplification from Nipponbare and then sub-cloned into the pCAMBIA1305.1-GUS vector. The resulting plasmid was transformed into rice through Agrobacterium-mediated transformation, and the transgenic plants were analyzed by GUS staining assay.

### RNA extraction and real-time RT-PCR analysis

Total RNA from roots, stems, leaves, leaf sheaths, panicles, and developing seeds at 6, 9, 12, 15, 18, 21, 24 DAF were extracted using the RNAprep pure Plant kit (TIANGEN Biotech, Beijing, China). 2 μg of total RNA was reverse-transcribed by priming with oligo(dT18) in a 40 μl reaction based on the PrimeScript Reverse Transcriptase kit (TaKaRa, http://www.takara-bio.com). Gene expression was measured by quantitative real-time PCR (qRT-PCR) using the *Ubiquitin* gene (GenBank accession AF184280) as reference. The qRT-PCR primers used to evaluate the expression of starch synthesis-related genes have been described previously^16^. PCR was performed with an ABI 7500 Fast Real-Time PCR System using the following program: 95 °C for 30 s, 40 cycles of 95 °C for 5 s, 60 °C for 34 s and 95 °C for 15 s. Changes in gene expression were calculated using the 2^−ΔΔCT^ method.

### Subcellular localization of OsBT1 protein

The *OsBT1* coding sequences without a termination codon of wild-type and *osbt1* were cloned in-frame in front of the GFP coding region in the TR2-GFP vector to create the fusion constructs TR2-OsBT1-GFP and TR2-osbt1-GFP. The two fusion constructs including the empty control vector (TR2-GFP) were introduced into the *Agrobacterium* strain EHA105 and used to infiltrate *N. benthamiana* leaves. Plants were then incubated at 28 °C for 2 days before examination according to protocols described previously^60^. *N. benthamiana* protoplasts were isolated as described previously^45^. GFP fluorescent signals were detected using a Zeiss LSM710 confocal laser scanning microscope. The *OsBT1* coding sequence without a termination codon was also cloned in-frame in front of GFP in the pCAMBIA1305-GFP vector to generate the 1305-OsBT1-GFP construct under the control of the maize *UBIQUTIN1* promoter. The 1305-OsBT1-GFP plasmid was transformed into an *indica* rice cv. Kitaake. Endosperms from T_1_ transgenic seeds were used for observation.

## Additional Information

**How to cite this article**: Li, S. *et al. OsBT1* encodes an ADP-glucose transporter involved in starch synthesis and compound granule formation in rice endosperm. *Sci. Rep.* **7**, 40124; doi: 10.1038/srep40124 (2017).

**Publisher's note:** Springer Nature remains neutral with regard to jurisdictional claims in published maps and institutional affiliations.

## Supplementary Material

Supplementary Information

## References

[b1] Lopez-Juez E. & Pyke K. A. Plastids unleashed: their development and their integration in plant development. Int. J. Dev. Biol. 49, 557–577 (2005). 16096965 10.1387/ijdb.051997el

[b2] Sakamoto W., Miyagishima S. Y. & Jarvis P. Chloroplast biogenesis: control of plastid development, protein import, division and inheritance. Arabidopsis Book. 6, e0110 (2008). 22303235 10.1199/tab.0110 PMC3243408

[b3] Martin C. & Smith A. M. Starch biosynthesis. Plant Cell. 7, 971–985 (1995). 7640529 10.1105/tpc.7.7.971 PMC160895

[b4] Hanashiro I. . Granule-bound starch synthase I is responsible for biosynthesis of extra-long unit chains of amylopectin in rice. Plant Cell Physiol. 49, 925–933 (2008). 18430767 10.1093/pcp/pcn066

[b5] James M. G., Robertson D. S. & Myers A. M. Characterization of the maize gene sugary1, a determinant of starch composition in kernels. Plant Cell. 7, 417–429 (1995). 7773016 10.1105/tpc.7.4.417 PMC160793

[b6] Ball S. . From glycogen to amylopectin: a model for the biogenesis of the plant starch granule. Cell. 86, 349–352 (1996). 8756717 10.1016/s0092-8674(00)80107-5

[b7] Myers A. M., Morell M. K., James M. G. & Ball S. G. Recent progress toward understanding biosynthesis of the amylopectin crystal. Plant Physiol. 122, 989–997 (2000). 10759494 10.1104/pp.122.4.989 PMC1539245

[b8] Lee S. K. . Identification of the ADPglucose pyrophosphorylase isoforms essential for starch synthesis in the leaf and seed endosperm of rice (*Oryza sativa L.*). Plant Mol. Biol. 65, 531–546 (2007). 17406793 10.1007/s11103-007-9153-z

[b9] Sano Y., Maekawa M. & Kikuchi H. Temperature effects on the Wx protein level and amylose content in the endosperm of rice. J. Hered. 76, 221–222 (1985).

[b10] Zhang M. Z. . Molecular insights into how a deficiency of amylose affects carbon allocation-carbohydrate and oil analyses and gene expression profiling in the seeds of a rice waxy mutant. BMC Plant Biol. 12, 230 (2012a). 23217057 10.1186/1471-2229-12-230 PMC3541260

[b11] Fujita N. . Characterization of SSIIIa-deficient mutants of rice: the function of SSIIIa and pleiotropic effects by SSIIIa deficiency in the rice endosperm. Plant Physiol. 144, 2009–2023 (2007). 17586688 10.1104/pp.107.102533 PMC1949899

[b12] Ryoo N. . Knockout of a starch synthase gene OsSSIIIa/Flo5 causes white-core floury endosperm in rice (*Oryza sativa L.*). Plant Cell Rep. 26, 1083–1095 (2007). 17297616 10.1007/s00299-007-0309-8

[b13] Kubo A. . The starch-debranching enzymes isoamylase and pullulanase are both involved in amylopectin biosynthesis in rice endosperm. Plant Physiol. 121, 399–410 (1999). 10517831 10.1104/pp.121.2.399 PMC59402

[b14] Kawagoe Y., Kubo A., Satoh H., Takaiwa F. & Nakamura Y. Roles of isoamylase and ADP-glucose pyrophosphorylase in starch granule synthesis in rice endosperm. Plant J. 42, 164–174 (2005). 15807780 10.1111/j.1365-313X.2005.02367.x

[b15] Nishi A., Nakamura Y., Tanaka N. & Satoh H. Biochemical and genetic analysis of the effects of amylose-extender mutation in rice endosperm. Plant Physiol. 127, 459–472 (2001). 11598221 PMC125082

[b16] She K. C. . A novel factor *FLOURY ENDOSPERM2* is involved in regulation of rice grain size and starch quality. Plant Cell. 22, 3280–3294 (2010). 20889913 10.1105/tpc.109.070821 PMC2990130

[b17] Peng C. . *FLOURY ENDOSPERM6* encodes a CBM48 domain-containing protein involved in compound granule formation and starch synthesis in rice endosperm. Plant J. 77, 917–930 (2014). 24456533 10.1111/tpj.12444

[b18] Wang E. . Control of rice grain-filling and yield by a gene with a potential signature of domestication. Nat. Genet. 40, 1370–1374 (2008). 18820698 10.1038/ng.220

[b19] Yang J. . Alanine aminotransferase 1 (OsAlaAT1) plays an essential role in the regulation of starch storage in rice endosperm. Plant Sci. 240, 77–89 (2015). 10.1016/j.plantsci.2015.07.027 26475189

[b20] Wang J. C., Xu H., Zhu Y., Liu Q. Q. & Cai X. L. OsbZIP58, a basic leucine zipper transcription factor, regulates starch biosynthesis in rice endosperm. J. Exp. Bot. 64, 3453–3466 (2013). 23846875 10.1093/jxb/ert187 PMC3733163

[b21] Fu F. F. & Xue H. W. Coexpression analysis identifies Rice Starch Regulator1, a rice AP2/EREBP family transcription factor, as a novel rice starch biosynthesis regulator. Plant Physiol. 154, 927–938 (2010). 20713616 10.1104/pp.110.159517 PMC2949045

[b22] Sullivan T. D., Strelow L. I., Illingworth C. A., Phillips R. L. & Nelson O. E. Analysis of maize *brittle-1* alleles and a defective *Suppressor-mutator*-induced mutable allele. Plant Cell. 3, 1337–1348 (1991). 1668652 10.1105/tpc.3.12.1337 PMC160096

[b23] Millar A. H. & Heazlewood J. L. Genomic and proteomic analysis of mitochondrial carrier proteins in Arabidopsis. Plant Physiol. 131, 443–453 (2003). 12586869 10.1104/pp.009985 PMC166821

[b24] Haferkamp I. The diverse members of the mitochondrial carrier family in plants. FEBS Lett. 581, 2375–2379 (2007). 17321523 10.1016/j.febslet.2007.02.020

[b25] Sullivan T. D. & Kaneko T. The maize *brittle1* gene encodes amyloplast membrane polypeptides. Planta. 196, 477–484 (1995). 7647682 10.1007/BF00203647

[b26] Fukao Y., Hayashi Y., Mano S., Hayashi M. & Nishimura M. Developmental analysis of a putative ATP/ADP carrier protein localized on glyoxysomal membranes during the peroxisome transition in pumpkin cotyledons. Plant Cell Physiol. 42, 835–841 (2001). 11522909 10.1093/pcp/pce108

[b27] Bouvier F. . Arabidopsis SAMT1 defines a plastid transporter regulating plastid biogenesis and plant development. Plant Cell. 18, 3088–3105 (2006). 17098813 10.1105/tpc.105.040741 PMC1693945

[b28] Palmieri L. . Molecular identification of an Arabidopsis S-adenosylmethionine transporter. Analysis of organ distribution, bacterial expression, reconstitution into liposomes, and functional characterization. Plant Physiol. 142, 855–865 (2006). 16950860 10.1104/pp.106.086975 PMC1630753

[b29] Kirchberger S., Tjaden J. & Neuhaus H. E. Characterization of the Arabidopsis Brittle 1 transport protein and impact of reduced activity on plant metabolism. Plant J. 56, 51–63 (2008). 18564385 10.1111/j.1365-313X.2008.03583.x

[b30] Kirchberger S. . Molecular and biochemical analysis of the plastidic ADP-glucose transporter (ZmBT1) from *Zea mays*. J. Biol. Chem. 282, 22481–22491 (2007). 17562699 10.1074/jbc.M702484200

[b31] Patron N. J. . The lys5 mutations of barley reveal the nature and importance of plastidial ADP-Glc transporters for starch synthesis in cereal endosperm. Plant Physiol. 135, 2088–2097 (2004). 15299120 10.1104/pp.104.045203 PMC520780

[b32] Bowsher C. G., Scrase-Field E. F. A. L., Esposito S., Emes M. J. & Tetlow I. J. Characterization of ADP-glucose transport across the cereal endosperm amyloplast envelope. J. Exp. Bot. 58, 1321–1332 (2007). 17301030 10.1093/jxb/erl297

[b33] Kang H. G., Park S., Matsuoka M. & An G. White-core endosperm floury endosperm-4 in rice is generated by knockout mutations in the C4-type pyruvate orthophosphate dikinase gene (OsPPDKB). Plant J. 42, 901–911 (2005). 15941402 10.1111/j.1365-313X.2005.02423.x

[b34] Bai A. J., Lu X. D., Li D. Q., Liu J. X. & Liu C. M. NF-YB1-regulated expression of sucrose transporters in aleurone facilitates sugar loading to rice endosperm. Cell Res. 26, 384–388 (2015). 26403192 10.1038/cr.2015.116 PMC4783462

[b35] Satoh H. & Omura T. New endosperm mutations induced by chemical mutagens in rice Oryza sativa L. Jap. J. Breed. 3, 316–326 (1981).

[b36] Nishio T. & Iida S. Mutants having a low content of 16-kDa allergenic protein in rice (*Oryza sativa L.*). Theor. Appl. Genet. 86, 317–321 (1993). 24193476 10.1007/BF00222095

[b37] Matsushima R., Maekawa M., Fujita N. & Sakamoto W. A rapid, direct observation method to isolate mutants with defects in starch grain morphology in rice. Plant Cell Physiol. 51, 728–741 (2010). 20360021 10.1093/pcp/pcq040

[b38] Wang Y. H. . OsRab5a regulates endomembrane organization and storage protein trafficking in rice endosperm cells. Plant J. 64, 812–824 (2010). 21105928 10.1111/j.1365-313X.2010.04370.x

[b39] Han X. H. . The failure to express a protein disulphide isomerase-like protein results in a floury endosperm and an endoplasmic reticulum stress response in rice. J. Exp. Bot. 63, 121–130 (2012). 21984651 10.1093/jxb/err262 PMC3245461

[b40] Zhang D. P., Wu J. G., Zhang Y. J. & Shi C. H. Phenotypic and candidate gene analysis of a new floury endosperm mutant (*osagpl2-3*) in rice. Plant Mol. Biol. Rep. 30, 1303–1312 (2012b).

[b41] Liu F. . OsVPS9A functions cooperatively with OsRAB5A to regulate post-Golgi dense vesicle-mediated storage protein trafficking to the protein storage vacuole in rice endosperm cells. Mol. Plant. 6, 1918–1932 (2013). 23723154 10.1093/mp/sst081

[b42] Matsushima R. . Amyloplast-localized SUBSTANDARD STARCH GRAIN4 protein influences the size of starch grains in rice endosperm. Plant Physiol. 164, 623–636 (2014). 24335509 10.1104/pp.113.229591 PMC3912094

[b43] Ren Y. L. . GLUTELIN PRECURSOR ACCUMULATION3 encodes a regulator of post-Golgi vesicular traffic essential for vacuolar protein sorting in rice endosperm. Plant Cell. 26, 410–425 (2014). 24488962 10.1105/tpc.113.121376 PMC3963586

[b44] Zhang L. . *FLOURY ENDOSPERM7* encodes a regulator of starch synthesis and amyloplast development essential for peripheral endosperm development in rice. J. Exp. Bot. 67, 633–647 (2015). 26608643 10.1093/jxb/erv469 PMC4737065

[b45] Matsushima R. . Amyloplast membrane protein SUBSTANDARD STARCH GRAIN6 controls Starch Grain Size in Rice Endosperm. Plant Physiol. 170, 1445–1459 (2016). 26792122 10.1104/pp.15.01811 PMC4775137

[b46] Duan E. C. . Pyrophosphate:fructose-6-phosphate1-phosphotransferase (PFP) regulates carbon metabolism during grain filling in rice. Plant Cell Rep. 6, 1321–1331 (2016). 10.1007/s00299-016-1964-4 PMC4869756 26993329

[b47] Toyosawa Y. . Deficiency of starch synthase IIIa and IVb alters starch granule morphology from polyhedral to spherical in rice endosperm. Plant Physiol. 170, 1255–1270 (2016). 26747287 10.1104/pp.15.01232 PMC4775109

[b48] Satoh H. T. . Mutation of the plastidial α-glucan phosphorylase gene in rice affects the synthesis and structure of starch in the endosperm. Plant Cell 20, 1833–1849 (2008). 18621947 10.1105/tpc.107.054007 PMC2518224

[b49] Zeng D. . *Du1*, encoding a novel Prp1 protein, regulates starch biosynthesis through affecting the splicing of *Wx*^*b*^ pre-mRNAs in rice (*Oryza sativa L.*). Plant Mol. Biol. 65, 501–509 (2007). 17579813 10.1007/s11103-007-9186-3

[b50] Isshiki M. . *Du3*, a mRNA cap-binding protein gene, regulates amylose content in Japonica rice seeds. Plant Biotechnol. 25, 483–487 (2008).

[b51] Li Y. . *Chalk5* encodes a vacuolar H^+^-translocating pyrophosphatase influencing grain chalkiness in rice. Nat. Genet. 46, 398–404 (2014). 24633159 10.1038/ng.2923

[b52] Delatte T., Trevisan M., Parker M. L. & Zeeman S. C. Arabidopsis mutants Atisa1 and Atisa2 have identical phenotypes and lack the same multimeric isoamylase, which influences the branch point distribution of amylopectin during starch synthesis. Plant J. 41, 815–830 (2005). 15743447 10.1111/j.1365-313X.2005.02348.x

[b53] Nakamura Y. . Characterization of the reactions of starch branching enzymes from rice endosperm. Plant Cell Physiol. 51, 776–794 (2010). 20305271 10.1093/pcp/pcq035

[b54] Satoh H. . Starch-branching enzyme I-deficient mutation specifically affects the structure and properties of starch in rice endosperm. Plant Physiol. 133, 1111–1121 (2003). 14526120 10.1104/pp.103.021527 PMC281607

[b55] Flügge U. I. Phosphate translocators in plastids. Annu. Rev. Plant Physiol. Plant Mol. Biol. 50, 27–45 (1999). 15012202 10.1146/annurev.arplant.50.1.27

[b56] Weber A. . Identification, purification, and molecular cloning of a putative plastidic glucose translocator. Plant Cell. 12, 787–802 (2000). 10810150 10.1105/tpc.12.5.787 PMC139927

[b57] Cakir B. . Analysis of the rice ADP-glucoes transporter (OsBT1) indicates the presence of regulatory processes in the amyloplast stroma that control ADP-glucose flux into starch. Plant Physiol. 170, 1271–1283 (2016). 26754668 10.1104/pp.15.01911 PMC4775147

[b58] Takemoto Y. . The rice mutant *esp2* greatly accumulates the glutelin precursor and deletes the protein disulfide isomerase. Plant Physiol. 128, 1212–1222 (2002). 11950970 10.1104/pp.010624 PMC154249

[b59] Liu L. . Identification and characterization of a novel Waxy allele from a Yunnan rice landrace. Plant Mol. Biol. 71, 609–626 (2009). 19760367 10.1007/s11103-009-9544-4

[b60] Chen S., Tao L., Zeng L., Vega-Sanchez M. E., Umemura K. & Wang G. L. A highly efficient transient protoplast system for analyzing defence gene expression and protein-protein interactions in rice. Mol. Plant. Pathol. 7, 417–427 (2006). 20507457 10.1111/j.1364-3703.2006.00346.x

