# Supplementary material for: OsBT1 encodes an ADP-glucose transporter involved in starch synthesis and compound granule formation in rice endosperm
Source: Sci Rep. 2017 Jan 5;7:40124. doi: 10.1038/srep40124 (PMC5215005; doi:10.1038/srep40124)
Supplement: Supplementary Information [file srep40124-s1.pdf]

# *OsBT1* encodes an ADP-glucose transporter involved in starch synthesis and compound granule formation in rice endosperm

Sanfeng Li [1,+], Xiangjin Wei [1,+], Yulong Ren [2], Jiehua Qiu [1], Guiai Jiao [1], Xiuping Guo [2], Shaoqing Tang [1], Jianmin Wan [2,*], Peisong Hu [1,*].

[1] State Key Laboratory of Rice Biology, China National Rice Research Institute, Hangzhou, 310006, China

[2] National Key Facility for Crop Resources and Genetic Improvement, Institute of Crop Science, Chinese Academy of Agricultural Sciences, Beijing 100081, PR China

[*] corresponding. peisonghu@126.com, hupeisong@caas.cn (P. Hu); wanjianmin@caas.cn (J. Wan)

[+] these authors contributed equally to this work.

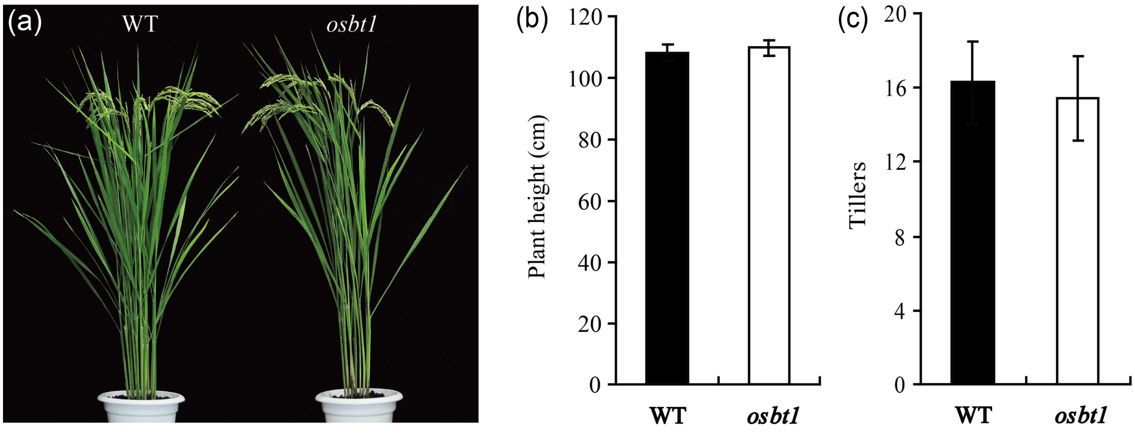


**Figure S1. Characteristics of adult wild-type and *osbt1* plants.**

(a) Comparison of wild-type and *osbt1* mutant plants after heading.

(b) Plant height of wild-type and *osbt1* mutant.

(c) Tiller number in wild-type and *osbt1* mutant. Values are means ±SD (n=20).

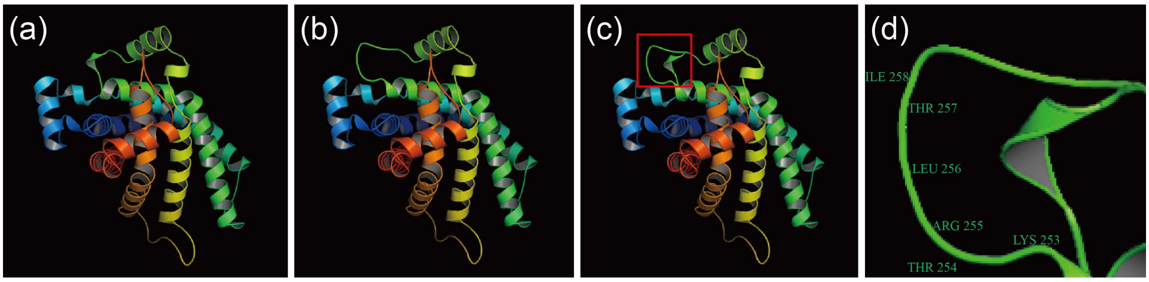


**Figure S2. Three-dimensional protein structures prediction.**

(a) OsBT1 three-dimensional structures prediction.

(b) osbt1 three-dimensional structures prediction.

(c) Merged (a) and (b).

(d) The differences areas between OsBT1 and osbt1 three-dimensional structures shown are indicated as red squares in (c). The extra six amino acids in osbt1 were marked.

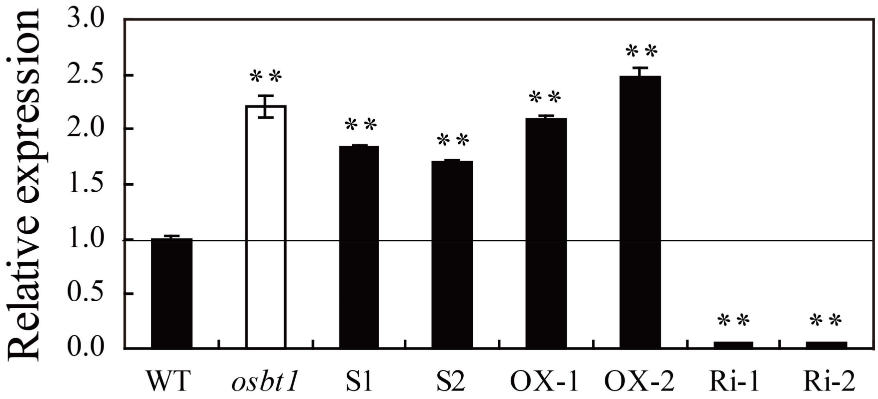


**Figure S3. Relative expression level of *OsBT1* in WT, *osbt1* and transgenic lines including complementary lines (S1, S2), overexpressing lines (OX-1, OX-2) and RNAi lines (Ri-1, Ri-2) at 15 DAF.**

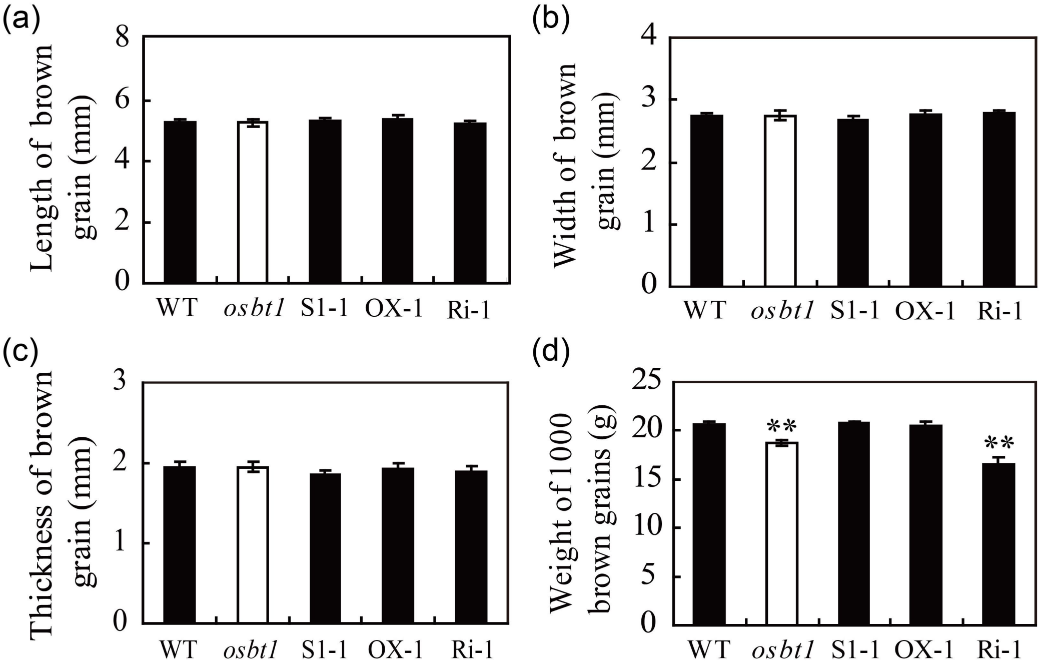


**Figure S4. Quantification of brown grain size and brown grain weight.**

(a) Brown grain length of WT, *osbt1*, complementary (S1-1), overexpressing (OX-1) and RNAi (Ri-1) lines.

(b) Brown grain width of WT, *osbt1*, complementary (S1-1), overexpressing (OX-1) and RNAi (Ri-1) lines.

(c) Brown grain thickness of WT, *osbt1*, complementary (S1-1), overexpressing (OX-1) and RNAi (Ri-1) lines.

(d) Weight of 1000 brown grains of WT, *osbt1*, complementary (S1-1), overexpressing (OX-1) and RNAi (Ri-1) lines.

Data are given as means ± SD from three replicates. Statistical comparisons were performed using Student's *t*-test; all data were compared with WT (*P < 0.05, **P < 0.01)

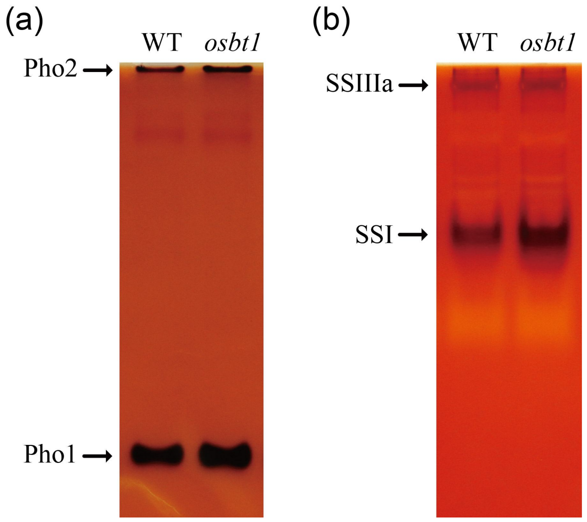


**Figure S5. Zymogram analysis of starch synthesis enzymes.**

Native-PAGE/activity staining analyses were performed using enzymes extracted from developing endosperms at 12DAF of the *osbt1* mutant and the wild-type.

(a) Starch phosphorylase activity staining for Pho1 and Pho2 (indicated by arrowheads).

(b) Starch synthase activity of SSI and SSIIIa.

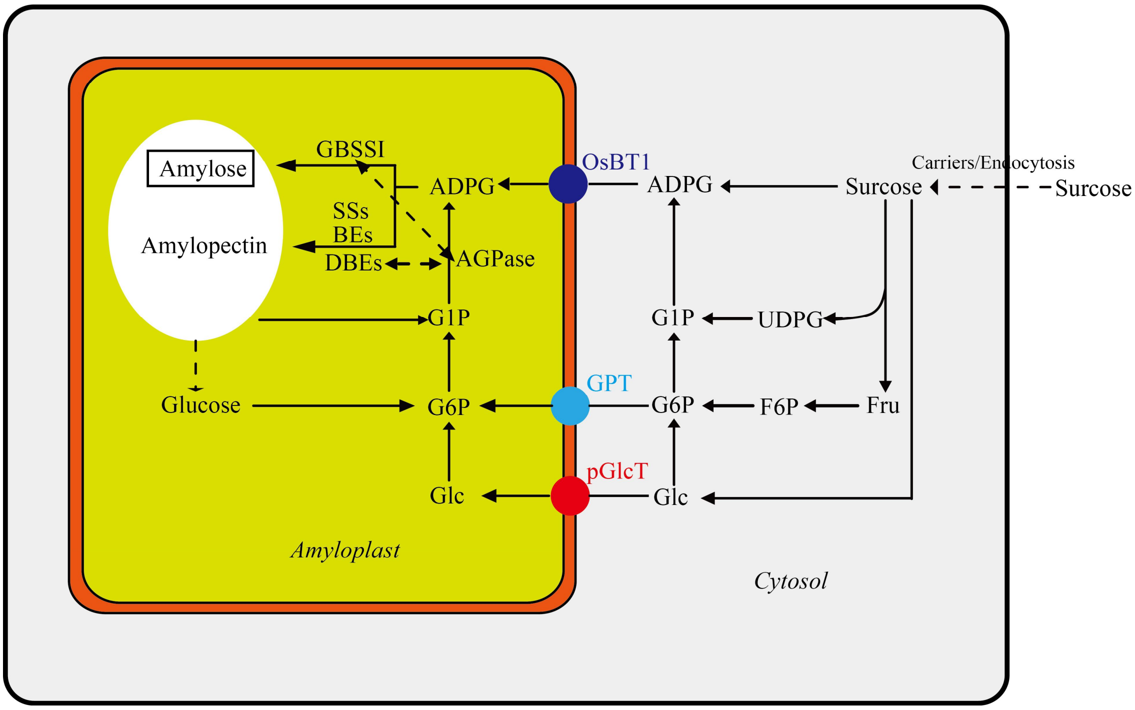


**Figure S6. Schematic representation of starch metabolism related to plastidic translocators for carbon in sink tissues of plants.**

ADPG, ADP-glucose; UDPG, UDP-glucose; G1P, glucose-1-phosphate; G6P, glucose-6-phosphate; F6P, fructose 6-phosphate; Fru, fructose; Glc, glucose; AGPase, ADPG pyrophosphorylase; GPT, glucose-6-phosphate translocator; pGlcT, plastidic glucose translocator.

**Table S1. Gene products of the seven predicted ORFs in the fine mapping region**

| ORFs | Gene Product Name |
|---|---|
| ORF1 | expressed protein |
| ORF2 | mitochondrial carrier protein, putative, expressed |
| ORF3 | protein of unknown function domain containing protein, expressed |
| ORF4 | Sel1 repeat domain containing protein, putative, expressed |
| ORF5 | serine acetyltransferase protein, putative, expressed |
| ORF6 | transposon protein, putative, unclassified, expressed |
| ORF7 | OsFBK3 - F-box domain and kelch repeat containing protein, expressed |

**Table S2. Primers used in this study**

| Use | Primer name | Sequence (5' to 3') |
|---|---|---|
| **Fine mapping** | RM12495-F | ACTACCTCCACTTCCTCCACTCC |
| | RM12495-R | GCTTAATGGTGGCGTCAAAGC |
| | RM5622-F | TCTTCGAAACAAGCAGCTCTAGG |
| | RM5622-R | GATAGGTCGGATGTGTGATTTGG |
| | RM12669-F | CCTGCAGTTGAGCAGCTTCTTCC |
| | RM12669-R | GCTCGTGCGCTACTCTTCTTGC |
| | RM12679-F | CTCGTCTCCTCATGCATATCAGC |
| | RM12679-R | TGCCTCGCTCTTGTCTTTACTCC |
| | RM5340-F | CGGACACCCAACGAGATGTACC |
| | RM5340-R | GGTCCTTGTCAACAATGCAAACC |
| | RM12671-F | ACGCTGTCAGTCACTTCCTCACC |
| | RM12671-R | CTATCTCATTATCGGCGATCACACC |
| | RM12674-F | TAAATGCCAACCAACTCCAAGC |
| | RM12674-R | AACTGCGTTTGGGAATATCTCG |
| | RM12676-F | ACTGACGATTGGGCACATTATTCC |
| | RM12676-R | CTGCAAATTGGTGGGTGATTGC |
| | Ind3-F | CAAGGCAATGGATTTGAA |
| | Ind3-R | GCTTTTGAGCCGAAAAGT |
| | Ind4-F | GGCTTAATAGTGTAAGAGTGG |
| | Ind4-R | ACCTGTTGATTCTGCATTG |
| | Ind10-F | CCCCTCTTTTCTTCATCAC |
| | Ind10-R | TATGGAGCAAGAACACCGA |
| **Genotyping** | Indel-F | GTGATCGAATCCATGGCGTC |
| | Indel-R | GGGGTTTGTGTGTGTTTGTG |
| | 2300-1F | TAGGCACCCCAGGCTTTACACT |
| | 2300-1R | AGCGGTTTTGGAAGGTGATTTG |
| **Binary vector construction** | 2300-OsBT1-EcoRI-F | CCATGATTACGAATTCGACGATGAGCCGGTAACAAT |
| | 2300-OsBT1-SmaI-R | CTCTAGAGGATCCCCGGGTCACAACAAACCCATCTCCA |
| | 1390-OsBT1-1F | TTTGGTACCGCATCAACAGCAGCAGCAAGAGG |
| | OsBT1-OX-F | GTAGAAGAGGTACCCGGGATGGCGGCGATGATGGCGGT |
| | OsBT1-OX-R | CTCTAGAGGATCCCCGGGTCACGCAACCTTCTCCTTGA |
| | 1390-OsBT1-1R | TTTGAGCTCCTCCGGGGTGAGGTACTTCTTGGCC |
| | 1390-OsBT1-2F | TTTGGATCCGCATCAACAGCAGCAGCAAGAGG |
| | 1390-OsBT1-2R | TTTCTGCAGCTCCGGGGTGAGGTACTTCTTGGCC |
| | pUbi-1305-OsBT1-GFP-BamHI-F | CGGTCCCGGGGGATCCATGGCGGCGATGATGGCGGT |
| | pUbi-1305-OsBT1-GFP-BamHI-R | TGCTCACCATGGATCCCGCAACCTTCTCCTTGATCT |
| | 1305-OsBT1-EcoRI-F | CCATGATTACGAATTCGACGATGAGCCGGTAACAAT |
| | 1305-OsBT1-NcoI-R | CTCAGATCTACCATGGCGATCACACCAAAATTAAAA |
| **Transient expression** | TR2-OsBT1-GFP-XbaI-F | CACCAAATCGTCTAGAATGGCGGCGATGATGGCGGT |
| | TR2-OsBT1-GFP-XbaI-R | TCGAGACGTCTCTAGACGCAACCTTCTCCTTGATCT |

| | | |
|---|---|---|
| **assays** | PAN580- OsBT1-GFP-SpeI-F | GCCCAGATCAACTAGTATGGCGGCGATGATGGCGGTG |
| | PAN580- OsBT1-GFP-XbaI-R | TCGAGACGTCTCTAGACGCAACCTTCTCCTTGATCTC |
| **Q-RT-PCR** | qRT-PCR-OsBT1-F | GGCATCTCCTTCATGTGCTA |
| | qRT-PCR-OsBT1-R | CGCAACCTTCTCCTTGATCT |
| | qRT-PCR-Ubi-F | GCTCCGTGGCGGTATCAT |
| | qRT-PCR-Ubi-R | CGGCAGTTGACAGCCCTAG |

**Table S3. Significance test of starch synthesis genes**

| Gene | Parents | 6 D | 9 D | 12 D | 15 D |
|---|---|---|---|---|---|
| | | Mean±SD | Mean±SD | Mean±SD | Mean±SD |
| *OsAGPL1* | WT | 1.0107±0.1851 | 0.1460±0.0142 | 0.4029±0.0093 | 0.9411±0.0248 |
| | *osbt1* | 0.9229±0.1716 | 0.2750±0.0321 | 0.6107±0.0618 | 1.6336±0.1736 |
| | *p*-Value | 0.5793 | 0.0031 | 0.0045 | 0.0024 |
| *OsAGPL2* | WT | 1.0001±0.0237 | 1.2910±0.0246 | 2.5470±0.0362 | 4.9466±0.0087 |
| | *osbt1* | 0.4890±0.0088 | 1.4455±0.0509 | 3.0844±0.1894 | 12.0664±0.3718 |
| | *p*-Value | <0.0001 | 0.0091 | 0.0085 | <0.0001 |
| *OsAGPS1* | WT | 1.0007±0.0526 | 0.8639±0.0447 | 0.9902±0.0120 | 1.1532±0.0350 |
| | *osbt1* | 4.3390±0.7012 | 3.2862±0.1340 | 1.5649±0.0807 | 1.2349±0.2770 |
| | *p*-Value | 0.0078 | <0.0001 | 0.0025 | 0.7192 |
| *OsAGPS2b* | WT | 1.0001±0.0193 | 1.7367±0.0897 | 2.9718±0.1347 | 3.7159±0.0849 |
| | *osbt1* | 0.5504±0.0273 | 3.2951±0.0891 | 4.1940±0.0849 | 9.0277±0.1546 |
| | *p*-Value | 0.0003 | <0.0001 | 0.0002 | <0.0001 |
| *OsBEI* | WT | 1.0000±0.0130 | 5.2539±0.0593 | 5.0288±0.1786 | 10.1073±0.0666 |
| | *osbt1* | 0.4171±0.0056 | 4.6804±0.2422 | 4.2692±0.0570 | 14.6747±0.1769 |
| | *p*-Value | <0.0001 | 0.0163 | 0.0022 | <0.0001 |
| *OsBEIIb* | WT | 1.0396±0.4019 | 2.8798±0.3989 | 10.8126±1.8399 | 11.9059±0.0478 |
| | *osbt1* | 1.7194±0.0292 | 2.5749±0.2406 | 15.7391±0.2152 | 19.3225±0.2353 |
| | *p*-Value | 0.1398 | 0.3203 | 0.0153 | 0.0005 |
| *OsSSI* | WT | 1.0029±0.0955 | 0.7807±0.0074 | 6.1889±0.1104 | 15.9733±0.3877 |
| | *osbt1* | 0.5626±0.0475 | 1.4779±0.0424 | 6.5403±0.1705 | 27.1463±2.3868 |
| | *p*-Value | 0.0017 | <0.0001 | 0.0401 | 0.0013 |
| *OsSSIIa* | WT | 1.0050±0.1254 | 0.9673±0.0117 | 1.3696±0.0195 | 2.7101±0.0037 |
| | *osbt1* | 0.4452±0.0037 | 1.3786±0.0604 | 1.6174±0.0432 | 5.0206±0.0706 |
| | *p*-Value | <0.0001 | 0.0028 | 0.0008 | <0.0001 |
| *OsSSIVb* | WT | 1.0043±0.1155 | 1.1535±0.0306 | 1.3764±0.0649 | 2.8458±0.1758 |
| | *osbt1* | 0.8360±0.0422 | 2.0149±0.0769 | 1.9944±0.0359 | 3.9931±0.1741 |
| | *p*-Value | 0.1049 | 0.0001 | 0.0001 | 0.0013 |
| *OsGBSSI* | WT | 1.0010±0.0565 | 1.8879±0.0179 | 2.7824±0.0760 | 7.8696±0.3881 |
| | *osbt1* | 1.2545±0.0060 | 3.4056±0.1616 | 6.4902±0.0471 | 14.7502±0.2108 |
| | *p*-Value | 0.0001 | 0.0001 | <0.0001 | <0.0001 |
| *OsISA1* | WT | 1.0038±0.1083 | 1.0864±0.0253 | 1.4504±0.0117 | 1.8399±0.0632 |
| | *osbt1* | 1.7658±0.2112 | 1.8453±0.0085 | 2.2193±0.0710 | 2.4934±0.2040 |
| | *p*-Value | 0.0138 | <0.0001 | 0.0001 | 0.0061 |
| *OsISA2* | WT | 1.0049±0.1247 | 2.3176±0.0261 | 4.4817±0.2070 | 9.2083±0.2889 |
| | *osbt1* | 0.6131±0.0085 | 2.4921±0.0152 | 4.5528±0.0961 | 16.1426±0.0368 |
| | *p*-Value | 0.0001 | 0.0037 | 0.6180 | 0.0001 |
| *OsPUL* | WT | 1.0055±0.1322 | 3.5868±0.3035 | 4.9785±0.0621 | 11.5173±0.0362 |
| | *osbt1* | 0.6075±0.0121 | 3.9660±0.0715 | 5.9085±0.2759 | 15.6721±0.5951 |
| | *p*-Value | 0.0002 | 0.1029 | 0.0047 | 0.0003 |

| | | | | | |
|---|---|---|---|---|---|
| *OsPHOL* | WT | 1.0059±0.1360 | 0.4046±0.0033 | 0.5183±0.0037 | 0.5986±0.0056 |
| | *osbt1* | 3.0087±0.0647 | 1.1482±0.0207 | 1.1301±0.0313 | 0.9767±0.0295 |
| | *p*-Value | <0.0001 | <0.0001 | <0.0001 | <0.0001 |

**Significance test of starch synthesis genes during seed development in WT and *osbt1* using Student's *t*-test. All data were given by mean±standard deviation (SD) (n = 3).**
